# Co-occurrence of tethered cord syndrome and cervical spine instability in hypermobile Ehlers-Danlos syndrome

**DOI:** 10.3389/fneur.2024.1441866

**Published:** 2024-07-17

**Authors:** Cortney Gensemer, Victoria Daylor, Jared Nix, Russell A. Norris, Sunil Patel

**Affiliations:** ^1^Department of Regenerative Medicine and Cell Biology, Medical University of South Carolina, Charleston, SC, United States; ^2^Department of Neurosurgery, Medical University of South Carolina, Charleston, SC, United States

**Keywords:** Ehlers Danlos syndrome, hypermobility, tethered cord syndrome, cervical instability, cranio-cervical junction, hypermobile Ehlers Danlos syndrome, occult tethered cord

## Abstract

The Ehlers-Danlos Syndromes (EDS) represent a group of hereditary connective tissue disorders, with the hypermobile subtype (hEDS) being the most prevalent. hEDS manifests with a diverse array of clinical symptoms and associated comorbidities spanning the musculoskeletal, neurological, gastrointestinal, cardiovascular, and immunological systems. hEDS patients may experience spinal neurological complications, including cervico-medullary symptoms arising from cranio-cervical and/or cervical instability/hypermobility, as well as tethered cord syndrome (TCS). TCS is often radiographically occult in nature, not always detectable on standard imaging and presents with lower back pain, balance issues, weakness in the lower extremities, sensory loss, and bowel or bladder dysfunction. Cervical instability due to ligament laxity can lead to headaches, vertigo, tinnitus, vision changes, syncope, radiculopathy, pain, and dysphagia. TCS and cervical instability not only share clinical features but can also co-occur in hEDS patients, posing challenges in diagnostics and clinical management. We present a review of the literature and a case study of a 20-year-old female with hEDS, who underwent surgical interventions for these conditions, highlighting the challenges in diagnosing and managing these complexities and underscoring the importance of tailored treatment strategies to improve patient outcomes.

## Introduction

### Ehlers-Danlos syndromes

The Ehlers-Danlos syndromes (EDS) encompass a spectrum of 14 heritable connective tissue disorders, characterized by varying degrees of joint hypermobility and tissue fragility (1, 2). Among these, hypermobile EDS (hEDS) is the most common subtype (3). Despite its prevalence, hEDS faces diagnostic hurdles stemming from a lack of discernable genetic causes and limited clinical recognition. Primary clinical signs of hEDS include joint hypermobility and instability, though individuals with hEDS often have co-morbid conditions beyond musculoskeletal issues, such as gastrointestinal, cardiac, immunological, neurological and dermatological manifestations (2, 4). The constellation of symptoms across multiple body systems, as well as chronic and acute pain, can make daily life challenging for this patient population. Craniospinal neurological manifestations affecting those with hEDS can include scoliosis, cerebrospinal fluid (CSF) leak, spinal instability, tethered cord syndrome and Chiari malformation among others (2, 4).

### Cranio-cervical and cervical spine instability

Instability or hypermobility of the cervical spine can result from trauma or ligament laxity. Instability can manifest in various ways, including cranio-cervical instability (CCI) at the junction between the skull and upper cervical spine, atlantoaxial instability (AAI) occurring at the upper cervical vertebrae (C1-C2), and lower cervical instability (CI) occurring below C2. Depending on the spinal level of instability, it can give rise to a range of neurological and musculoskeletal symptoms including headache, fatigue, vertigo, tinnitus, vision changes, syncope and pre-syncope, hyperreflexia, gait changes, limb pain and /or weakness, radiculopathy-myelopathy, dystonia, neck and facial pain and dysphagia (5, 6). Complications such as vertebral artery kinking, autonomic dysfunction and compromised vertebral blood and/or CSF flow may occur (5, 7, 8). Symptoms of cervical spine instability may overlap with other conditions, including Chiari malformation, therefore both conditions should be considered in the diagnostic process when evaluating hEDS patients presenting with the above symptoms. The prevalence of CCI/CI in the hEDS patient population may be as high as 31.6%, although the prevalence in the general population is unknown (4).

Evaluation of instability can be challenging with supine, static imaging. Hypermobility or instability typically becomes apparent on dynamic imaging performed in the upright position, as the signs and symptoms of instability often emerge when the lax ligaments are stressed by the head’s weight under gravity. This position can cause dynamic effects on the underlying neural structures, such as the medulla and spinal cord, leading to issues like medullary kinking or spinal cord stretching (7, 9). Three measurements that are used to evaluate for CCI on radiographic imaging are the clivoaxial angle, the horizontal Harris measurement (Basion-Axis interval), and the Grabb-Mapstone-Oakes measurement (6, 7, 9, 10). Assessing for AAI in imaging involves looking at rotation of C1 on C2, vertical displacement (Chamberlain, McRae, and McGregor lines) and horizontal displacement (atlantodens interval) (8, 9). Segmental cervical instability, below the level of C2, is often, but not always, associated with spondylosis and disc degeneration (8). In addition, the loss of cervical lordosis (or kyphosis) is often noted. In the event of unremarkable upright dynamic imaging, abnormal spinal cord motion may still be present at the cranio-cervical junction in hEDS patients, causing symptoms consistent with CCI (11).

Treatment of cervical spine instabilities varies based on the severity of symptoms and radiologic findings. It’s important to note that radiological findings do not always correspond to clinical manifestations, and individuals with hEDS may have multiple levels affected, including CCI, AAI, and segmental instabilities. Conservative approaches include immobilizing the neck with a cervical collar, rest, stopping activities that exacerbate symptoms and physical therapy focusing on isometric exercises with an experienced therapist (8, 12). If non-operative approaches fail, occipito-cervical or cervical fusion with instrumentation for stabilization of the affected levels may be required (7, 8, 13).

### Tethered cord syndrome

Tethered cord syndrome (TCS) results from restricted movement of the spinal cord. The caudal end of the spinal cord (conus medullaris) is attached to a fibrous band of connective tissue called the filum terminale (FT) interna, which, in a healthy individual, acts as a buffer system, protecting the spinal cord from traction (14). In individuals with TCS, the FT undergoes abnormal changes, becoming either rigid, enlarged with fibrous or fatty tissue (known as a “thick” or “fatty” filum respectively), and/or exhibiting a low-lying conus medullaris. The inelastic properties of the FT in TCS result in limited movement of the spinal cord, placing stress on the conus medullaris. A cadaver study of healthy and TCS FT showed evidence that stress was translated, not only to the conus medullaris, but to the lumbar spinal cord, and that the FT, together with the dentate ligament, create a protective force of the cranial segments of the spinal cord (15). This tension on the conus and spinal cord can manifest as symptoms of lower back pain (aching and/or burning), lower extremity weakness and or pain, balance problems, sensory loss, heaviness, leg cramps, and parasethsias (8, 16). These clinical findings are often asymmetrical (8). Additional features of TCS include bowel and/or bladder incontinence, urinary frequency, urinary hesitancy, sexual dysfunction, nocturia, frequent urinary tract infections, and constipation, among others (8, 16). Neurological exam findings can help determine the level of spinal stress, with the presence of foot clonus, increased leg tone, and increased lower-extremity hyperreflexia indicative of upper motoneuron dysfunction (17). The prevalence of TCS in the general population is unknown, but it is estimated to be 6.65% in individuals hEDS based on a study involving 2,149 clinically diagnosed hEDS patients who completed a self-reported survey focusing on diagnostic and comorbid conditions (4). This estimate is likely conservative due to challenges in diagnosing TCS and the limited awareness and literature specifically addressing the association between TCS and hEDS.

TCS can be classified into two types: classical and occult. Classical TCS typically presents between infancy and childhood with a low laying conus (below vertebral L2-L3) or a fatty/thickened FT seen on imaging (15, 18). A variety of other congenital conditions categorized into this type of tethering are radiographically apparent, including diastematomyelia, myelomeningoceles, lipomas, among others. Occult tethered cord syndrome is characterized by clinical complaints consistent with classical TCS, yet imaging demonstrates a normal position of the conus (8). Until better diagnostic tools are available, a clinical diagnosis of occult TCS must be made based on the neurological symptoms described above, in which the combination of symptoms may vary between patients. In addition, urodynamic testing can be utilized to determine aspects of a neurogenic bladder related to TCS, although this testing is not required to make an occult TCS diagnosis (8). MRI of the whole spine is recommended to rule out other neurological conditions that cause back pain and leg weakness (8). The standard approach to treat occult TCS is lumbar laminectomy and transection/resection of the FT. Although it is not common, it is important to be aware of the possibility that patients may “re-tether” after surgical intervention. If patients experience recurrent symptoms after surgery, it’s crucial to consider the possibility of re-tethering and the impact on conditions like CCI and cervical instability (13, 19). The neurological presentations of hEDS are complex, overlapping, and pose diagnostic and treatment challenges. Acknowledging and addressing the concurrent occurrence of TCS and CCI/CI in individuals with hEDS will contribute to enhanced outcomes and an improved quality of life.

## Case report

**Figure 1:**
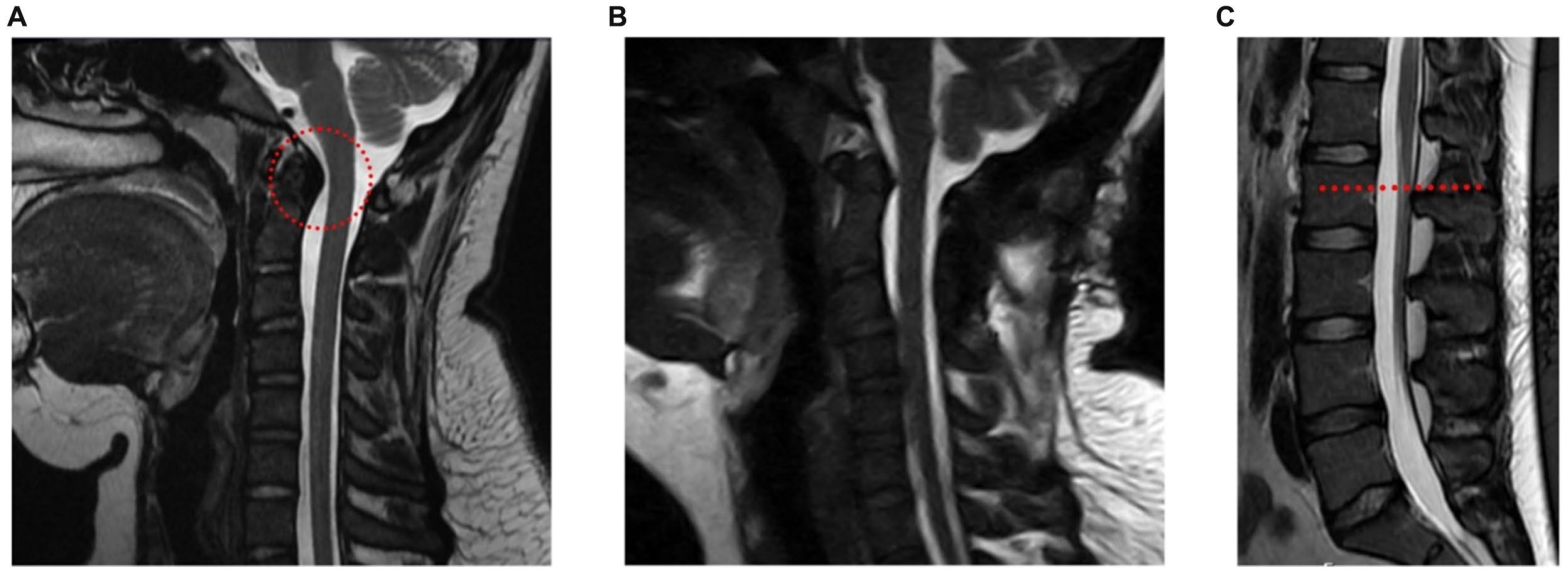
A young woman with hEDS, CCI, and TCS. **(A)** Upright cervical flexion MRI showing medullary kinking (red circle) (clivo-axial angle of 116 degrees). **(B)** Postoperative upright MRI showing reduced medullary kinking after fixation and fusion. **(C)** MRI of lumbar spine showing a slightly low-lying conus at mid L2 body (red line).

A 20-year-old female presented in the outpatient neurosurgery clinic with a history of postural orthostatic hypotension syndrome (POTS) and hEDS. At initial evaluation, she presented with symptoms including severe daily suboccipital headaches, neck pain, and myelopathy, despite prior unremarkable supine cervical MRI. Myelopathic complaints included leg weakness and gait imbalance. Exam findings consistent with myelopathy included increased patellar reflexes, poor tandem step gait, increased tone in the leg muscles and positive Rhomberg sign. These neurological symptoms led to significant limitations in her daily activities, ultimately leading the patient to withdraw from college, as she could no longer attend classes. Physical therapy (PT) had been attempted intermittently for 3 years prior with a therapist familiar with hEDS. The patient reported dramatic improvement in symptoms while wearing a rigid cervical collar, though benefit was short lasting after removal of her collar. An upright cervical flexion/extension MRI confirmed a diagnosis of craniocervical instability, showing clivo-axial angle in flexion of 116 degrees (clivo-axial angle of <130 degrees indicates pathologic) and medullary kinking (Figure 1A). Due to the progressive symptoms and resulting disability, she was offered an occipital cervical fusion (C0 to C3) to correct her craniocervical instability. This case involved the use of an occipital plate, C2 pedicle and C3 facet screws with fixation and hinged rods, as well as bone allograft for arthrodesis.

The surgery was successful (Figure 1B), although recovery measured over the following year was delayed due to a fall at home in the immediate postoperative period, though imaging confirmed stable hardware and early bony fusion. After restarting PT and weaning the use of her collar as needed, she found significant improvement in her daily headaches, neck pain, myelopathic complaints, as well as subjective improvement in her POTS and dysautonomia symptoms. Over time the patient experienced intermittent periods of headache exacerbations. She revisited PT and implemented short-term activity modifications, which led to improvement of headaches with decreasing frequency and severity over time. She continued to report non-dermatomal upper extremity paresthesias, however overall functional limitation from myelopathy had resolved. She was able to return to university, graduate and began working as a licensed social worker.

Approximately four years later, the patient presented with complaints of non-dermatomal bilateral lower extremities (BLE) numbness and pain, as well as urinary dysfunction (frequency and retention). Her urinary symptoms did not respond to 4 years of attempted pelvic floor therapy. The urinary complaints began as a young child, in which she experienced urge incontinence and retention. She had worn urinary pads for many years, which eventually progressed to the point of using an intermittent catheter (self-catheterization) at least once a day, and urodynamic studies confirmed a neurogenic bladder. She experienced constant low back pain and had not responded to PT or medication management. BLE paresthesias were consistent with increased neural tension, most often improved by lying in the fetal position and keeping knees bent when resting, consistent with TCS symptoms in adults.

A lumbar MRI at the time was reported unremarkable with her conus medullaris ending at mid L2, slightly lower than typical L1/L2, but not radiographically obvious like the presentation of classical TCS (Figure 1C). The symptoms and imaging are consistent with radiographically occult TCS. The patient underwent a L1-L2 partial laminectomy for tethered cord release utilizing microsurgical technique, which involves partial removal of the associated spinous processes and interspinous ligament. Following adequate visualization of the dura, an incision was made allowing for CSF egress and careful examination of the intradural space. The filum was then identified and a portion of it just below the conus was resected. Meticulous attention was placed on achieving watertight closure of the durotomy with gortex sutures. The patient is placed on bed rest with a subfascial drain in place for at least 24 h.

She tolerated this procedure well, and within the first month she noted early improvement in her urinary symptoms as well as her lower extremity paresthesias. Her low back pain gradually improved over 3 months following completion of postoperative PT. Urinary symptoms improved, no longer experiencing retention nor need to use a self-catheter. The reduction in the above symptoms allowed her to return to work fully after a 3-month recovery. At the 6-month postoperative appointment, the patient reported some remaining lower right leg parasethias, resolved lower back and sacral pain, and 90% resolution of preoperative incontinence and intermittent urinary symptoms.

## Discussion

**Figure 2:**
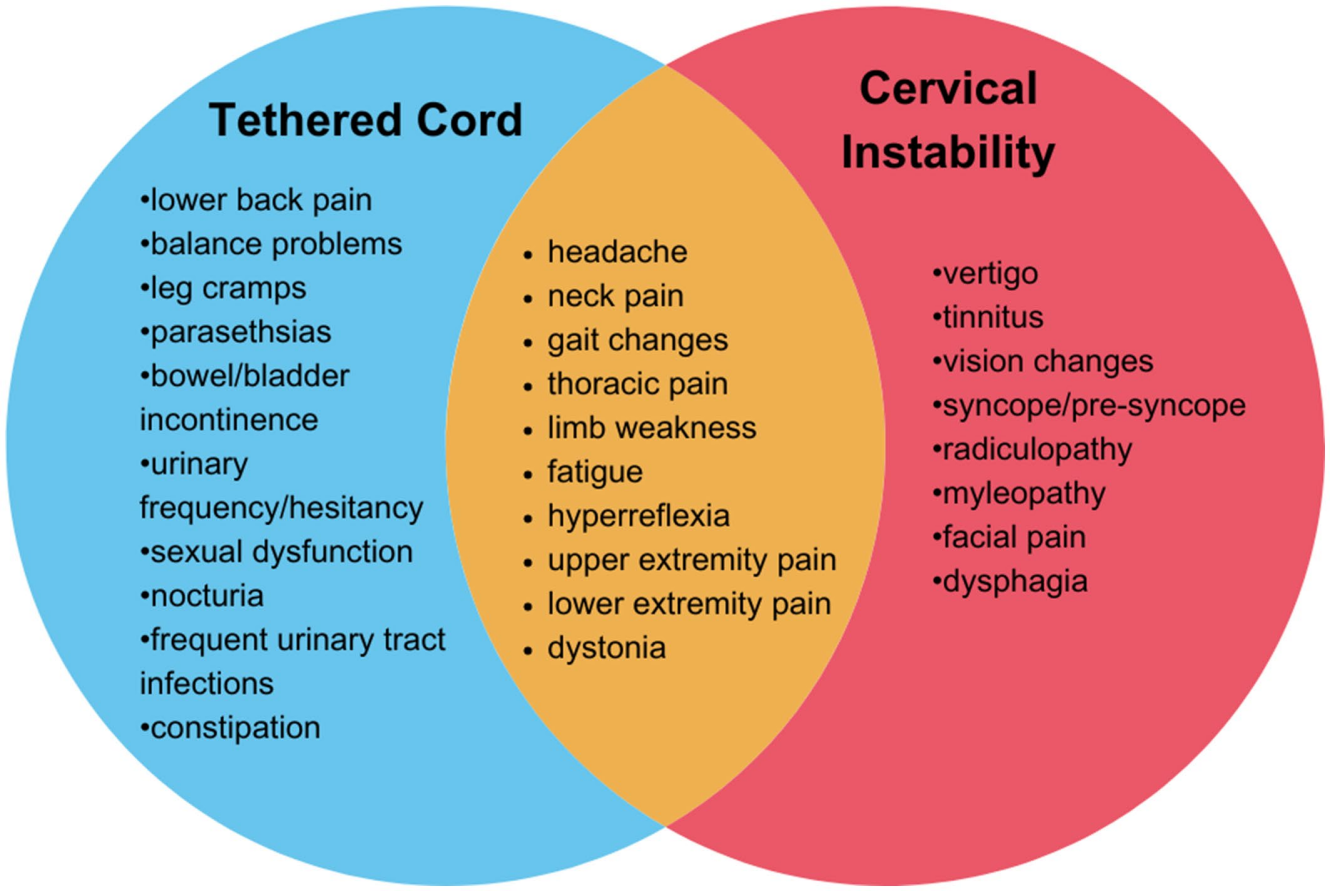
Overlapping symptoms and neurological findings of tethered cord syndrome and cervical instability. Tethered cord syndrome (blue), cervical instability (red) overlapping features in both conditions (orange).

hEDS is a complex connective tissue disorder characterized by joint hypermobility and tissue fragility, often accompanied by a range of co-existing conditions that complicate diagnosis and treatment. hEDS patients may present with co-existing tethered cord syndrome and cranio-cervical and /or cervical instability, or symptoms consistent with both (Figure 2). A recent study of 2,149 individuals diagnosed with hEDS identified three distinct phenotypic clusters, one of which (comprising 11.5% of the cohort) showed an increased prevalence of spinal and neurological manifestations and a disease burden of over 14 conditions (4). In a retrospective study including patients with an EDS diagnosis whom also underwent neurosurgery (n = 67), 17.9% had a diagnosis of tethered cord syndrome (TCS) and 85.1% had a diagnosis of craniocervical instability and/or atlantoaxial instability (CCI/AAI) (13). It is important for healthcare providers to recognize these symptoms and determine the most effective treatment strategies.

Spinal instability and TCS are likely underdiagnosed in the hEDS population, as is hEDS itself. The patient described had symptoms for nearly 15 years before she was diagnosed with hEDS. Her spinal symptoms lead to standard imaging of the spine in the supine position, and these were initially reposted ‘normal’, until she had upright dynamic imaging of the spine. While the patient did manifest signs and symptoms of a myelopathy, it was not until she had upright imaging of the cranio/cervical spine that the confirmation was made of instability. TCS in this patient population also appears to be under-diagnosed. A detailed history of pain, weakness and urinary functions can often alert the provider to the possibility of occult TCS in the context of hEDS, as highlighted in this case study.

Thus far, surgical intervention has proven a successful course of treatment. In our clinic, treatment of TCS prior to cervical fusion for cervical instability is recommended, because of the possible improvement in cervical symptom severity once the downward tension of the spinal cord is released. In the case study, the patient was not treated for TCS as symptoms were not obvious until after cervical fusion, however addressing TCS prior to fusion may have improved some of her cervical symptoms, possibly eliminating the need for fusion altogether. Surgical intervention of TCS is remarkably of low morbidity, estimated in our clinic as 2 months, compared to cervical fusion for hEDS patients, estimated as 6 months, especially when considering non-surgical treatment options are available for instability. Additionally, surgical intervention for instability requires permanent hardware, or additional surgeries to remove the implanted hardware, adding to the total recovery time, pain, and interference with the patient’s quality of life. Outcomes of cranio-cervical fixation for CCI in EDS patients are encouraging (6, 20), but the risk of hardware or fusion failure is elevated in individuals with EDS (21). In addition to these risks, fusion also raises concerns for adjacent level disease (22). While limited studies are available, surgical outcomes for tethered cord in EDS are generally positive although the occurrence of re-tethering is a possibility (6, 17, 23). A recent publication of occult TCS used a 15-item scale to indicate clinical criteria for surgical intervention, showing clinical improvement in 89% of patients at 3-month follow up and 68% of patients at 12-month follow up, as well as accurately predicting the outcome of surgical intervention in 82% of cases. Using the 15-item scale, it was observed that patients with a preoperative greater than 8, the likelihood of surgical improvement exceeded 80%, indicating the utility of this scale as a valuable clinical tool (22). Another study reported surgical outcomes of filum sectioning in EDS patient with TCS were comparable or greater than surgical intervention for classical TCS patients (23). Specifically, improvements have been reported in ambulatory abilities, low back pain, and urinary symptoms (9). Co-morbid conditions also warrant special consideration in treatment strategies, especially in regard to mast cell activation disorder (MCAD) and autonomic dysfunction.

The underlying pathophysiology of spinal instability in hEDS patients is thought to be due to ligament laxity in structures supporting the head, spinal cord and neck (6, 11). In contrast, in TCS hEDS patients have impaired elasticity of the FT tissues, resulting in increased mechanical forces that are transmitted to the conus medularis (23). Additionally, hEDS-associated FT demonstrates disorganized collagen fibrils, inflammatory cell infiltration, and heightened susceptibility to mechanical stress (23). This prompts the question of whether tension exerted on the spinal cord worsens cervico-medullary symptoms due to instability. Furthermore, given the co-occurrence of these conditions in hEDS patients, it raises questions into whether stiffening of the FT acts as a compensatory or protective mechanism against spinal laxity and excess cord motion. Patients falling into hEDS cluster with higher incidence of instability and TCS also have higher rates of MCAD, which may align with the inflammatory infiltration reported in TCS and could be contributing to disease pathogenesis (4, 23).

Further research is needed to identify mechanisms of the progression and onset of spinal instabilities and TCS in hEDS patients. Investigations should prioritize innovative diagnostic methodologies, optimal treatment modalities, surgical interventions, and strategies to mitigate TCS recurrence. Beyond neurological manifestations, the underlying genetic and biological mechanisms driving hEDS remain elusive. Enhanced understanding of these mechanisms and their interplay with neurological manifestations and comorbidities is essential for improving diagnosis and management of individuals with hEDS.

## Data availability statement

The original contributions presented in the study are included in the article/supplementary material, further inquiries can be directed to the corresponding author.

## Ethics statement

Ethical approval was not required for the study involving humans in accordance with the local legislation and institutional requirements. Written informed consent to participate in this study was not required from the participants or the participants’ legal guardians/next of kin in accordance with the national legislation and the institutional requirements. Written informed consent was obtained from the individual(s) for the publication of any potentially identifiable images or data included in this article.

## Author contributions

CG: Visualization, Writing – original draft, Writing – review & editing. VD: Visualization, Writing – original draft, Writing – review & editing. JN: Data curation, Methodology, Writing – review & editing. RN: Supervision, Writing – review & editing. SP: Data curation, Methodology, Supervision, Writing – review & editing.
